# SGLT2is vs. GLP1RAs Reduce Cardiovascular and All-Cause Mortality

**DOI:** 10.3389/fcvm.2021.791311

**Published:** 2021-12-07

**Authors:** Mei Qiu, Xu-Bin Wei, Wei Wei

**Affiliations:** ^1^Department of General Medicine, Shenzhen Longhua District Central Hospital, Shenzhen, China; ^2^Department of Cardiology, The Second Affiliated Hospital of Kunming Medical University, Kunming, China; ^3^Department of Pharmacy, The Second Affiliated Hospital of Kunming Medical University, Kunming, China

**Keywords:** GLP1Ras, type 2 diabetes, death, cardiovascular, renal, SGLT2is

## Abstract

Lin et al. recently did a network meta-analysis based on cardiovascular (CV) outcome trials (CVOTs) of sodium-glucose cotransporter 2 inhibitors (SGLT2is) and those of glucagon-like peptide-1 receptor agonists (GLP1RAs). Due to the absence of CVOTs directly comparing SGLT2is with GLP1RAs, Lin et al.'s network meta-analysis identified the indirect evidence that SGLT2is vs. GLP1RAs reduced hospitalization for heart failure (HHF) but did not reduce CV death and all-cause mortality (ACM) in patients with type 2 diabetes (T2D). We did another meta-analysis incorporating those CV outcome cohort studies directly comparing SGLT2is with GLP1RAs, and identified that SGLT2is vs. GLP1RAs were significantly associated with the lower risks of not only HHF but also CV death and ACM. These findings may suggest that SGLT2is should be considered over GLP1RAs in terms of preventing CV and all-cause death and HHF in T2D patients.

## Introduction

We read with great interest Lin et al.'s network meta-analysis (1) recently published in the journal *Diabetologia*. By performing network meta-analysis based on 21 placebo-controlled cardiovascular (CV) outcome trials (CVOTs), Lin et al. (1) yielded the estimators for the relative cardiorenal efficacy of three new classes of hypoglycemic drugs: sodium-glucose cotransporter 2 inhibitors (SGLT2is), glucagon-like peptide-1 receptor agonists (GLP1RAs), and dipeptidyl peptidase-4 inhibitors. They identified that SGLT2is vs. GLP1RAs reduced hospitalization for heart failure (HHF) and composite kidney outcome (CKO), but did not reduce CV death [risk ratio (RR) 0.97, 95% confidence interval (CI) 0.87–1.09] and all-cause mortality (ACM) (RR 0.97, 95% CI 0.88–1.08). Due to in Lin et al.' article (1) the effect estimators among active drugs deriving from indirect evidence, the relative efficacy of SGLT2is vs. GLP1RAs revealed by Lin et al. (1) requires to be confirmed by further studies directly comparing these two classes, as stated in the last paragraph of Lin et al.' article (1). Hence, we included CV outcome cohort studies directly comparing SGLT2is with GLP1RAs, due to the absence of CVOTs directly comparing them, to conduct another meta-analysis to determine the relative efficacy of SGLT2is vs. GLP1RAs on CV death and ACM as well as other cardiorenal outcomes.

## Methods

This meta-analysis was conducted according to the preferred reporting items for systematic reviews and meta-analyses (PRISMA) statement (2). Its study protocol had been registered in PROSPERO (Registration number: CRD42021273721) before the study selection began. The studies eligible for inclusion were propensity score-matched (PSM) cohort studies which compared SGLT2is with GLP1RAs in terms of the effects of cardiorenal endpoints in patients with type 2 diabetes (T2D). Seven endpoints of interest were CV death, ACM, HHF, CKO, major adverse cardiovascular events (MACE), myocardial infarction (MI), and stroke. Composite kidney outcome and MACE were defined in detail in study protocol. PubMed and Embase were searched until August 16th 2021 to identify relevant cohort studies. The search terms mainly used in this meta-analysis were: “type 2 diabetes,” “T2D,” “sodium-glucose transporter 2 inhibitors,” “SGLT^*^,” “glucagon-like peptide-1 receptor agonists,” “GLP1^*^,” “death,” “mortality,” “cardiovascular,” “CKD,” “renal,” and “PSM.” Two authors independently assessed included studies for quality according to the Newcastle-Ottawa Scale (NOS) for cohort studies (3). Any agreements between them were addressed by discussion with a third author. We performed random-effects meta-analysis with the maximum likelihood method using hazard ratios (HRs) and 95% CIs derived from included articles. *I*^2^ statistic was calculated to measure heterogeneity. All data analyses were done in Stata/MP (version 16.0).

## Results

In this meta-analysis we included 9 large PSM cohort studies (4–12). Each of included studies was assessed as high quality according to NOS. The detailed characteristics of included studies are shown in Supplementary Table 1, which also provides the outcome data extracted from included articles. Meta-analysis involving 93,710 SGLT2is users and 94,935 GLP1RAs users from seven trials showed that SGLT2is and GLP1RAs had similar risk of MACE (HR 0.97, 95% CI 0.93–1.02; P for drug effect =0.24; Figure 1.1). Meta-analysis involving 93,710 SGLT2is users and 94,935 GLP1RAs users from seven trials showed that SGLT2is and GLP1RAs had similar risk of MI (HR 0.95, 95% CI 0.88–1.03; P for drug effect = 0.22; Figure 1.2). Meta-analysis involving 93,710 SGLT2is users and 94,935 GLP1RAs users from seven trials showed that SGLT2is and GLP1RAs had similar risk of stroke (HR 1.02, 95% CI 0.94–1.11; P for drug effect = 0.65; Figure 1.3). Meta-analysis involving 62,419 SGLT2is users and 63,644 GLP1RAs users from three trials showed that SGLT2is vs. GLP1RAs were significantly associated with an 18% reduction in risk of CV death (HR 0.82, 95% CI 0.68–0.99; P for drug effect = 0.04; Figure 1.4). Meta-analysis involving 101,636 SGLT2is users and 97,703 GLP1RAs users from six trials showed that SGLT2is vs. GLP1RAs were significantly associated with an 8% reduction in risk of ACM (HR 0.92, 95% CI 0.85–0.99; P for drug effect = 0.03; Figure 1.5). Meta-analysis involving 107,858 SGLT2is users and 107,563 GLP1RAs users from 8 trials showed that SGLT2is vs. GLP1RAs were significantly associated with a 20% reduction in risk of HHF (HR 0.80, 95% CI 0.70–0.92; P for drug effect < 0.01; Figure 1.6). Meta-analysis involving 30,641 SGLT2is users and 33,395 GLP1RAs users from two trials showed that SGLT2is and GLP1RAs had similar risk of CKO (HR 0.97, 95% CI 0.92–1.02; P for drug effect = 0.25; Figure 1.7).

**Figure 1:**
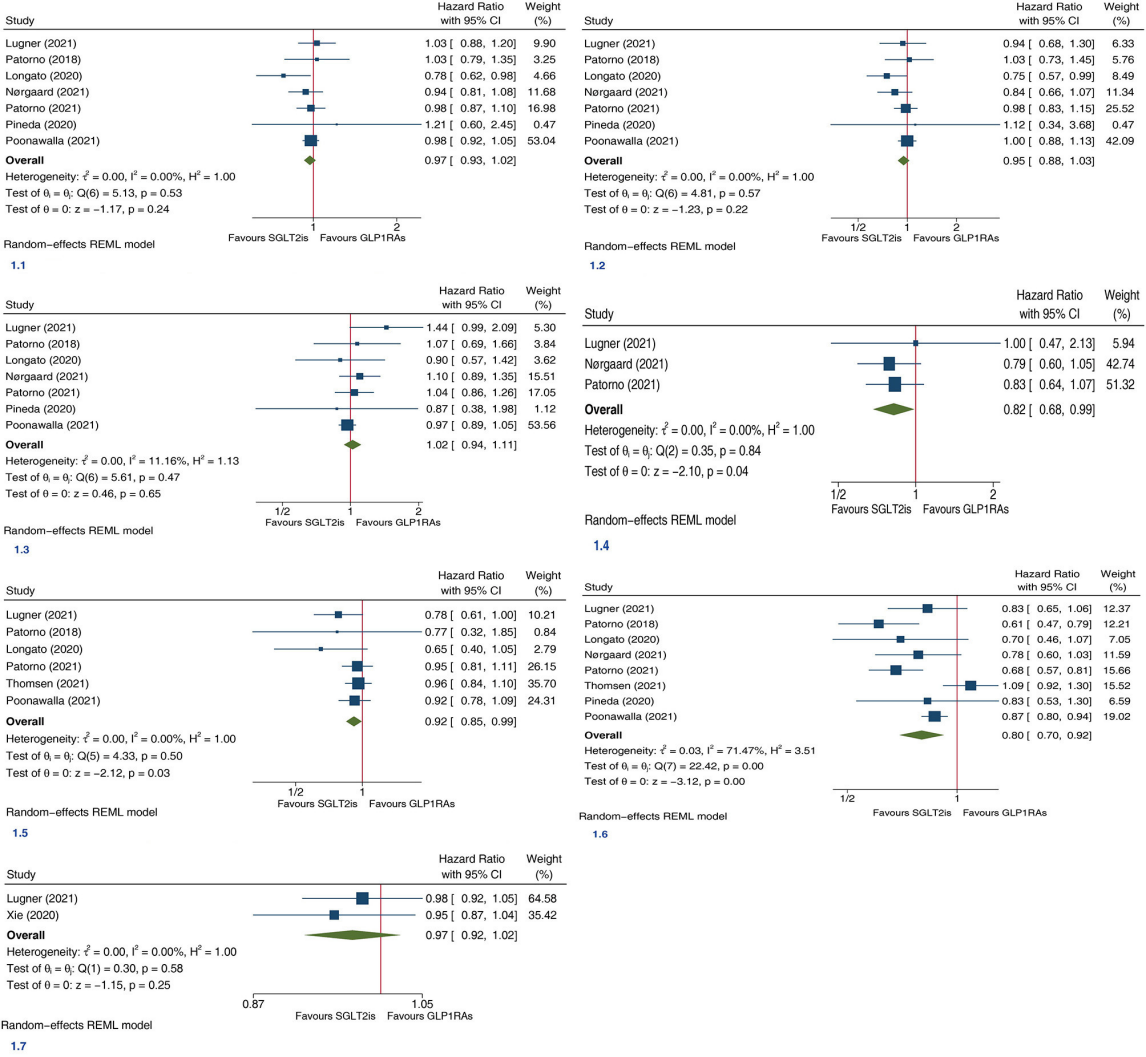
Forest plots of the efficacy of SGLT2is vs. GLP1RAs on seven (**1.1**, Major adverse cardiovascular events; **1.2**, myocardial infarction; **1.3**, stroke; **1.4**, cardiovascular death; **1.5**, all-cause mortality; **1.6**, hospitalization for heart failure; **1.7**, composite kidney outcome) cardiorenal and death endpoints in patients with type 2 diabetes. SGLT2is, sodium-glucose cotransporter 2 inhibitors; GLP1RAs, glucagon-like peptide-1 receptor agonists; CI, confidence interval; REML, maximum likelihood method.

## Discussion

This meta-analysis is the first one that provided the direct evidence regarding the relative efficacy of SGLT2is vs. GLP1RAs on death and cardiorenal endpoints in T2D patients by incorporating large PSM cohort studies directly comparing SGLT2is with GLP1RAs. Consistent with the indirect evidence from Lin et al.'s network meta-analysis (1), the direct evidence in our meta-analysis showed that SGLT2is vs. GLP1RAs significantly reduced HHF, but did not significantly affect MACE, MI, and stroke. On the contrary, the indirect evidence from Lin et al.'s network meta-analysis (1) showed that SGLT2is vs. GLP1RAs significantly reduced CKO, whereas the direct evidence in our meta-analysis showed that SGLT2is and GLP1RAs had the similar risk of CKO. The reason for this probably is that our meta-analysis was not powered to assess CKO since only two studies were included in the pooled analysis for this outcome. Most importantly, Lin et al.'s meta-analysis (1) failed to reveal the significantly reduced risks of CV death and ACM with SGLT2is vs. GLP1RAs in T2D patients, whereas our meta-analysis revealed those. Possible reasons are as follows. First, Lin et al.'s meta-analysis (1) gave the indirect evidence whereas ours gave the direct evidence. Second, for these two death outcomes our meta-analysis was with greater statistical power since cohort studies included in this meta-analysis had greater sample sizes than CVOTs. Third, our meta-analysis was based on HRs, whereas Lin et al.'s meta-analysis (1) was based on RRs. Compared to RRs, HRs additionally contain the information of the time when events happen apart from the information of whether events happen.

From 2015 to 2021, published are eight CVOTs (13–20) targeting the relative efficacy of GLP1RAs vs. placebo on cardiorenal outcomes in T2D patients. Although most of these CVOTs demonstrated the obvious benefits of GLP1RAs vs. placebo on CV composite and/or renal composite outcomes, none of them was powered enough to assess individual critical endpoints such as CV death and ACM. Therefore, several meta-analyses (21–23) based on the CVOTs of GLP1RAs were conducted to have confirmed the relative benefits of GLP1RAs compared to placebo on various cardiorenal outcomes including the above two death endpoints in T2D patients. Similar with GLP1RAs, SGLT2is were confirmed, by relevant meta-analyses (24–26) based on their CVOTs, with the distinct benefits on multiple cardiorenal and mortality endpoints compared to placebo in T2D patients. Accordingly, the latest international consensus report (27) by the American Diabetes Association (ADA) and the European Association for the Study of Diabetes (EASD) recommends that both SGLT2is and GLP1RAs should be used in T2D patients with established CV or renal disease and in those at high cardiorenal risk to prevent cardiorenal events and deaths. It is worth mentioning that the cardiorenal benefits of SGLT2is have extended from T2D patients to renal failure patients and heart failure patients including those with heart failure and a reduced ejection fraction and those with heart failure and a preserved ejection fraction. On the contrary, the cardiorenal benefits of GLP1RAs are only limited to T2D patients, and their cardiorenal benefits have not been observed in patients with renal or heart failure without T2D until now.

Due to the absence of CVOTs comparing GLP1RAs and SGLT2is, the relative efficacy of these two drug classes on cardiorenal endpoints is not given in the ADA and EASD consensus report (27). Thus, several network meta-analyses (1, 28, 29) including Lin et al.'s network meta-analysis (1) tried to derive the estimators of their relative cardiorenal efficacy by incorporating the indirect evidence from placebo-controlled CVOTs of GLP1RAs and those of SGLT2is. However, the different characteristics of those CVOTs included in the network meta-analyses (1, 28, 29) considerably weakened the credibility of the indirect evidence regarding the relative cardiorenal efficacy of GLP1RAs and SGLT2is. In contrast, more reliable is the direct evidence regarding their relative cardiorenal efficacy deriving from this present meta-analysis based on large PSM cohort studies directly comparing SGLT2is with GLP1RAs in terms of cardiorenal endpoints. Different from Lin et al.'s findings (1) that SGLT2is vs. GLP1RAs reduced HHF and CKO, but did not reduce CV death and ACM in T2D patients, our findings are that SGLT2is vs. GLP1RAs were significantly associated with the lower risks of not only HHF but also CV death and ACM. These findings may suggest that SGLT2is should be considered over GLP1RAs in terms of preventing CV and all-cause death and HHF in T2D patients.

SGLT2is and GLP1RAs exert their glycemic control effects via different mechanisms of actions: SGLT2is promote urinary glucose excretion (30), while GLP1RAs enhance insulin secretion and suppress glucagon secretion. Moreover, both of these two drug classes have favorable effects on some cardiometabolic risk factors such as blood pressure and body weight. More importantly, the long-term cardiorenal benefits exhibited by them are almost independent of their hypoglycemic effects. SGLT2is exert the long-term cardiorenal benefits mainly by improving mitochondrial function and myocardial efficiency, and reducing oxidative stress, inflammation, fibrosis, and sympathetic nervous system activation (31); while GLP1RAs exert these benefits mainly by improving endothelial function, reducing oxidative stress and vascular inflammation, and producing a natriuretic and vasodilator effect (32, 33). Besides, the benefits of these two drug classes on cardiometabolic risk factors also contribute to their benefits on long-term cardiorenal endpoints. Among the mechanisms of improving long-term cardiorenal prognosis, there are some similar mechanisms for these two drug classes, whereas there are more different mechanisms for them. Since those different mechanisms for SGLT2is and GLP1RAs might be complementary, the combination therapy of SGLT2is and GLP1RAs might yield more cardiorenal benefits than SGLT2is or GLP1RAs monotherapy. Future randomized CVOTs assessing this kind of combination therapy will be clinically meaningful.

Compared to previous network meta-analyses (1, 28, 29) based on those placebo-controlled CVOTs of SGLT2is and GLP1RAs, our meta-analysis is the first one that provided the direct evidence regarding the relative cardiorenal efficacy of SGLT2is vs. GLP1RAs. Compared to the eligible cohort studies included in our meta-analysis, this meta-analysis study has the following two strengths. First, included cohort studies produced many inconsistent findings. For example, some of the included cohort studies showed the significant association of SGLT2is with lower risks of HHF and MACE compared to GLP1RAs, whereas others showed that these two drug classes had similar risks of HHF and MACE. In contrast, this meta-analysis study addressed these controversies. Second, none of the included cohort studies revealed the significant association of SGLT2is with lower risks of CV death and ACM compared to GLP1RAs, which suggested the limited statistical power for these two death endpoints among included cohort studies. In contrast, this meta-analysis study, with the sufficient statistical power, revealed SGLT2is with significantly lower risks of CV death and ACM.

Although this meta-analysis provided the direct evidence regarding the relative cardiorenal efficacy of SGLT2is vs. GLP1RAs, the evidence derived from cohort studies, which involve more risks of biases than randomized trials do. Although the cohort studies included in this meta-analysis performed PSM analysis to adjusted lots of confounding factors, there were probably some omissive factors. Thus, there is a need for CVOTs comparing SGLT2is with GLP1RAs in T2D patients, to further confirm the direct evidence revealed in this meta-analysis. Although no substantial heterogeneity was observed in the meta-analyses on most of the endpoints assessed in this study, the substantial heterogeneity (*I*^2^ = 71.47%) was observed in the meta-analysis on HHF. Although we utilized the random-effects model to derive the conservative pooled results, it would be beneficial that future studies could perform relevant subgroup analyses for this outcome to explore the sources of heterogeneity.

## Conclusion

Lin et al. (1) revealed that SGLT2is vs. GLP1RAs significantly reduced HHF and CKO, but did not reduce CV death and ACM in T2D patients, whereas we further revealed that SGLT2is vs. GLP1RAs were significantly associated with the lower risks of not only HHF but also CV death and ACM. These findings may suggest that SGLT2is should be considered over GLP1RAs in terms of preventing CV and all-cause death and HHF in T2D patients, although further validation by CVOTs directly comparing SGLT2is with GLP1RAs in T2D patients would be beneficial.

## Data Availability Statement

The original contributions presented in the study are included in the article/Supplementary Material, further inquiries can be directed to the corresponding author/s.

## Author Contributions

MQ: design and writing manuscript. MQ, X-BW, and WW: conduct, data collection, and analysis. All authors contributed to the article and approved the submitted version.

## Conflict of Interest

The authors declare that the research was conducted in the absence of any commercial or financial relationships that could be construed as a potential conflict of interest.

## Publisher's Note

All claims expressed in this article are solely those of the authors and do not necessarily represent those of their affiliated organizations, or those of the publisher, the editors and the reviewers. Any product that may be evaluated in this article, or claim that may be made by its manufacturer, is not guaranteed or endorsed by the publisher.

## Supplementary Material

Supplementary Table 1 Characteristics of included studies and extracted data.

## Abbreviations

SGLT2is: sodium-glucose cotransporter 2 inhibitors
GLP1RAs: glucagon-like peptide-1 receptor agonists
CVOTs: cardiovascular outcome trials
T2D: type 2 diabetes
PSM: propensity score-matched
RR: risk ratio
HR: hazard ratio
CI: confidence interval
MACE: major adverse cardiovascular events
HHF: hospitalization for heart failure
CV: cardiovascular
MI: myocardial infarction
ACM: all-cause mortality
CKO: composite kidney outcome
PRISMA: preferred reporting items for systematic reviews and meta-analyses
NOS: Newcastle-Ottawa Scale
ADA: American Diabetes Association
EASD: European Association for the Study of Diabetes.

## References

[1] Lin DS Lee JK Hung CS Chen WJ. The efficacy and safety of novel classes of glucose-lowering drugs for cardiovascular outcomes: a network meta-analysis of randomised clinical trials. Diabetologia. (2021) 64:2676–86. 10.1007/s00125-021-05529-w 34536085

[2] Moher D Liberati A Tetzlaff J Altman DG. Preferred reporting items for systematic reviews and meta-analyses: the PRISMA statement. PLoS Med. (2009) 6:e1000097. 10.1371/journal.pmed.1000097 19621072 PMC2707599

[3] Stang A. Critical evaluation of the Newcastle-Ottawa Scale for the assessment of the quality of nonrandomized studies in meta-analyses. Eur J Epidemiol. (2010) 25:603–5. 10.1007/s10654-010-9491-z 20652370

[4] Patorno E Pawar A Bessette LG Kim DH Dave C Glynn RJ. Comparative effectiveness and safety of sodium-glucose cotransporter 2 inhibitors versus glucagon-like peptide 1 receptor agonists in older adults. Diabetes Care. (2021) 44:826–35. 10.2337/dc20-1464 33495295 PMC7896266

[5] Nørgaard CH Starkopf L Gerds TA Vestergaard P Bonde AN Fosbøl E. Cardiovascular outcomes with GLP-1 receptor agonists versus SGLT-2 inhibitors in patients with type 2 diabetes. Eur Heart J Cardiovasc Pharmacother. (2021) 2021:pvab053. 10.1093/ehjcvp/pvab053 34215881

[6] Poonawalla IB Bowe AT Tindal MC Meah YA Schwab P. A real-world comparison of cardiovascular, medical and costs outcomes in new users of SGLT2 inhibitors versus GLP-1 agonists. Diabetes Res Clin Pract. (2021) 175:108800. 10.1016/j.diabres.2021.108800 33845052

[7] Lugner M Sattar N Miftaraj M Ekelund J Franzén S Svensson AM. Cardiorenal and other diabetes related outcomes with SGLT-2 inhibitors compared to GLP-1 receptor agonists in type 2 diabetes: nationwide observational study. Cardiovasc Diabetol. (2021) 20:67. 10.1186/s12933-021-01258-x 33752680 PMC7983265

[8] Thomsen RW Knudsen JS Kahlert J Baggesen LM Lajer M Holmgaard PH. Cardiovascular events, acute hospitalizations, and mortality in patients with type 2 diabetes mellitus who initiate empagliflozin versus liraglutide: a comparative effectiveness study. J Am Heart Assoc. (2021) 10:e19356. 10.1161/JAHA.120.019356 34032121 PMC8483550

[9] Longato E Di Camillo B Sparacino G Gubian L Avogaro A Fadini GP. Cardiovascular outcomes of type 2 diabetic patients treated with SGLT-2 inhibitors versus GLP-1 receptor agonists in real-life. BMJ Open Diabetes Res Care. (2020) 8:e001451. 10.1136/bmjdrc-2020-001451 32591373 PMC7319723

[10] Xie Y Bowe B Gibson AK McGill JB Maddukuri G Yan Y. Comparative effectiveness of SGLT2 inhibitors, GLP-1 receptor agonists, DPP-4 inhibitors, and sulfonylureas on risk of kidney outcomes: emulation of a target trial using health care databases. Diabetes Care. (2020) 43:2859–69. 10.2337/dc20-1890 32938746

[11] Pineda ED Liao IC Godley PJ Michel JB Rascati KL. Cardiovascular outcomes among patients with type 2 diabetes newly initiated on sodium-glucose cotransporter-2 inhibitors, glucagon-like peptide-1 receptor agonists, and other antidiabetic medications. J Manag Care Spec Pharm. (2020) 26:610–8. 10.18553/jmcp.2020.26.5.610 32347181 PMC10391160

[12] Patorno E Goldfine AB Schneeweiss S Everett BM Glynn RJ Liu J. Cardiovascular outcomes associated with canagliflozin versus other non-gliflozin antidiabetic drugs: population based cohort study. BMJ. (2018) 360:k119. 10.1136/bmj.k119 29437648 PMC5799855

[13] Gerstein HC Sattar N Rosenstock J Ramasundarahettige C Pratley R Lopes RD. Cardiovascular and renal outcomes with efpeglenatide in type 2 diabetes. N Engl J Med. (2021) 385:896–907. 10.1056/NEJMoa2108269 34215025

[14] Holman RR Bethel MA Mentz RJ Thompson VP Lokhnygina Y Buse JB. Effects of once-weekly exenatide on cardiovascular outcomes in type 2 diabetes. N Engl J Med. (2017) 377:1228–39. 10.1056/NEJMoa1612917 28910237 PMC9792409

[15] Pfeffer MA Claggett B Diaz R Dickstein K Gerstein HC Kober LV. Lixisenatide in patients with type 2 diabetes and acute coronary syndrome. N Engl J Med. (2015) 373:2247–57. 10.1056/NEJMoa1509225 26630143

[16] Marso SP Daniels GH Brown-Frandsen K Kristensen P Mann JF Nauck MA. Liraglutide and cardiovascular outcomes in type 2 diabetes. N Engl J Med. (2016) 375:311–22. 10.1056/NEJMoa1603827 27295427 PMC4985288

[17] Marso SP Bain SC Consoli A Eliaschewitz FG Jodar E Leiter LA. Semaglutide and cardiovascular outcomes in patients with type 2 diabetes. N Engl J Med. (2016) 375:1834–44. 10.1056/NEJMoa1607141 27633186

[18] Hernandez AF Green JB Janmohamed S D'Agostino RS Granger CB Jones NP. Albiglutide and cardiovascular outcomes in patients with type 2 diabetes and cardiovascular disease (harmony outcomes): a double-blind, randomised placebo-controlled trial. Lancet. (2018) 392:1519–29. 10.1016/S0140-6736(18)32261-X 30291013

[19] Gerstein HC Colhoun HM Dagenais GR Diaz R Lakshmanan M Pais P. Dulaglutide and cardiovascular outcomes in type 2 diabetes (REWIND): a double-blind, randomised placebo-controlled trial. Lancet. (2019) 394:121–30. 10.1016/S0140-6736(19)31149-3 31189511

[20] Husain M Birkenfeld AL Donsmark M Dungan K Eliaschewitz FG Franco DR. Oral semaglutide and cardiovascular outcomes in patients with type 2 diabetes. N Engl J Med. (2019) 381:841–51. 10.1056/NEJMoa1901118 31185157

[21] Kristensen SL Rorth R Jhund PS Docherty KF Sattar N Preiss D. Cardiovascular, mortality, and kidney outcomes with GLP-1 receptor agonists in patients with type 2 diabetes: a systematic review and meta-analysis of cardiovascular outcome trials. Lancet Diabetes Endocrinol. (2019) 7:776–85. 10.1016/S2213-8587(19)30249-9 31422062

[22] Giugliano D Maiorino MI Bellastella G Longo M Chiodini P Esposito K. GLP-1 receptor agonists for prevention of cardiorenal outcomes in type 2 diabetes: an updated meta-analysis including the REWIND and PIONEER 6 trials. Diabetes Obes Metab. (2019) 21:2576–80. 10.1111/dom.13847 31373167

[23] Sattar N Lee M Kristensen SL Branch K Del PS Khurmi NS. Cardiovascular, mortality, and kidney outcomes with GLP-1 receptor agonists in patients with type 2 diabetes: a systematic review and meta-analysis of randomised trials. Lancet Diabetes Endocrinol. (2021) 9:653–62. 10.1016/S2213-8587(21)00203-5 34425083

[24] Zelniker TA Wiviott SD Raz I Im K Goodrich EL Bonaca MP. SGLT2 inhibitors for primary and secondary prevention of cardiovascular and renal outcomes in type 2 diabetes: a systematic review and meta-analysis of cardiovascular outcome trials. Lancet. (2019) 393:31–9. 10.1016/S0140-6736(18)32590-X 30424892

[25] Neuen BL Young T Heerspink H Neal B Perkovic V Billot L. SGLT2 inhibitors for the prevention of kidney failure in patients with type 2 diabetes: a systematic review and meta-analysis. Lancet Diabetes Endocrinol. (2019) 7:845–54. 10.1016/S2213-8587(19)30256-6 31495651

[26] Qiu M Ding L Zhou H. Effects of SGLT2 inhibitors on cardiovascular and renal outcomes in type 2 diabetes: a meta-analysis with trial sequential analysis. Medicine (Baltimore). (2021) 100:e25121. 10.1097/MD.0000000000025121 33725910 PMC7969215

[27] Buse JB Wexler DJ Tsapas A Rossing P Mingrone G Mathieu C. 2019 update to: Management of hyperglycaemia in type 2 diabetes, 2018. A consensus report by the American Diabetes Association (ADA) and the European Association for the Study of Diabetes (EASD). Diabetologia. (2020) 63:221–8. 10.1007/s00125-019-05039-w 31853556

[28] Fei Y Tsoi MF Cheung B. Cardiovascular outcomes in trials of new antidiabetic drug classes: a network meta-analysis. Cardiovasc Diabetol. (2019) 18:112. 10.1186/s12933-019-0916-z 31462224 PMC6714383

[29] Alfayez OM Al YM Alshibani M Fallatah SB Al KN Alsheikh R. Network meta-analysis of nine large cardiovascular outcome trials of new antidiabetic drugs. Prim Care Diabetes. (2019) 13:204–11. 10.1016/j.pcd.2019.01.003 30713085

[30] Heerspink HJ Perkins BA Fitchett DH Husain M Cherney DZ. Sodium glucose cotransporter 2 inhibitors in the treatment of diabetes mellitus: cardiovascular and kidney effects, potential mechanisms, and clinical applications. Circulation. (2016) 134:752–72. 10.1161/CIRCULATIONAHA.116.021887 27470878

[31] Zelniker TA Braunwald E. Mechanisms of cardiorenal effects of sodium-glucose cotransporter 2 inhibitors: JACC state-of-the-art review. J Am Coll Cardiol. (2020) 75:422–34. 10.1016/j.jacc.2019.11.031 32000955

[32] Savarese G Butler J Lund LH Bhatt DL Anker SD. Cardiovascular effects of non-insulin glucose-lowering agents: a comprehensive review of trial evidence and potential cardioprotective mechanisms. Cardiovasc Res. (2021) 2021:cvab271. 10.1093/cvr/cvab271 34390570

[33] Wilcox T De Block C Schwartzbard AZ Newman JD. Diabetic agents, from metformin to SGLT2 inhibitors and GLP1 receptor agonists: JACC focus seminar. J Am Coll Cardiol. (2020) 75:1956–74. 10.1016/j.jacc.2020.02.056 32327107 PMC7219531

